# Analyzing the Influence of Sizing Machine Settings on the Performance of the Sized Cotton Warp Yarns Using Eco-Friendly Carboxymethyl Cellulose from Saudi Wheat Straw

**DOI:** 10.3390/polym18101262

**Published:** 2026-05-21

**Authors:** Samah Maatoug, Elham Abu Nab

**Affiliations:** Department of Fashion and Textiles Design, Faculty of Art and Design, University of Tabuk, Tabuk 71491, Saudi Arabia

**Keywords:** sizing, wheat straw, carboxymethyl cellulose, wet zone yarn tension, squeezing pressure, sized yarn, sizing machine speed

## Abstract

CMC_ws_ is a low-cost, biodegradable carboxymethyl cellulose derived from Saudi wheat straw (CMC_ws_) as a sustainable alternative to traditional sizing agents for cotton warp yarns. The effects of key sizing parameters—wet zone yarn tension (350–410 N), squeezing pressure (220–330 N/m), and machine speed (30–70 m/min)—on the weavability performance of CMC_ws_-sized yarns were investigated by analyzing size add-on, tensile properties, hairiness, and abrasion resistance of sized warp yarns. Response surface methodology (RSM) based on a Box–Behnken experimental design comprising 15 runs was employed to optimize the machine settings and processing parameters for CMCws-sized yarns. Increasing wet zone yarn tension and squeezing pressure reduced size add-on and elongation at break, whereas higher sizing machine speed increased size add-on. Squeezing pressure showed a strong positive influence on abrasion resistance and adhesion power, while yarn hairiness increased with wet zone yarn tension and sizing machine speed. Maximum size add-on occurred at 70 m/min, 220 N/m, and 380 N, whereas optimum abrasion resistance was obtained at around 340 N, 330 N/m, and 45 m/min. Numerical optimization predicted minimum hairiness at about 350 N, 320 N/m, and 50 m/min. Overall, optimized settings significantly enhanced yarn mechanical performance and weavability, confirming CMC_ws_ as an effective, eco-friendly sizing agent for sustainable textile processing.

## 1. Introduction

Weaving continues to be the dominant technique in textile manufacturing, with woven fabrics maintaining a significantly larger share of the market compared with knitted and nonwoven structures [1,2,3]. However, over the last four decades, rapid growth in knitting and nonwoven technologies has intensified competition for the weaving sector. Improving weaving efficiency and fabric quality, therefore, relies heavily on effective preparation processes, among which warp yarn sizing is particularly crucial [4].

Sizing is a key operation in weaving preparation, involving the application of a thin adhesive film onto the yarn surface to enable the warp to withstand the mechanical stresses and strains encountered during weaving. In addition to increasing yarn strength, sizing improves abrasion resistance, and modern high-speed weaving is practically impossible without it [5,6]. After weaving, however, the applied size must be readily removable during desizing, since it is no longer required in the final fabric [2,7].

Among lignocellulosic resources, wheat straw represents an abundant and underutilized agricultural residue. In Saudi Arabia, annual wheat production is approximately 700,000 tons, generating substantial quantities of straw. It has been reported that 1 kg of wheat grain yields about 1.3–1.4 kg of straw. Wheat straw contains about 34.6–41.4% cellulose and roughly 72% holocellulose, confirming its suitability as a feedstock for biopolymer synthesis and value-added applications. As a renewable raw material, cellulose can be chemically modified into water-soluble derivatives, among which carboxymethyl cellulose (CMC) is produced by partial substitution of cellulose hydroxyl groups with ionic hydrophilic carboxymethyl groups [7].

The performance stability of wheat-straw-derived carboxymethyl cellulose (CMC_ws_) is crucial for its sustainable industrial use as a bio-based sizing agent. Important characteristics such as degree of substitution, solution viscosity, and molecular-weight distribution depend on the inherent variability of lignocellulosic feedstocks arising from harvest season, geographic origin, and agricultural conditions. Such variability can influence sizing behavior, yarn weavability, and process efficiency [7]. In the present work, wheat straw was sourced from a single harvest and region to minimize variability and enable systematic evaluation of CMC_ws_ sizing performance on cotton yarns. This controlled approach facilitates reproducible, low-impact sizing formulations and supports the development of quality-control criteria necessary for scaling wheat-straw-based CMC within a circular bioeconomy that valorizes agricultural residues and reduces reliance on fossil-derived sizing materials.

The quality of sized yarn depends on numerous processing variables, including yarn tension, squeezing pressure, rubber-roller hardness, machine speed, size viscosity, size-box temperature, and immersion-roller depth [2,5,8]. During weaving, warp yarns undergo complex mechanical actions such as abrasion, cyclic bending, impact, and tension. Therefore, controlling yarn structural parameters and mechanical properties, together with evaluating weavability, is essential.

Size add-on is an important key parameter affecting weavability. It is influenced by factors such as squeeze pressure, paste viscosity, machine speed, yarn tension, size-box level, immersion depth, yarn twist, count, and fiber type. In this study, yarn tension, squeeze pressure, and machine speed were selected as the principal variables. Squeeze pressure governs the penetration of the size paste into the yarn structure and removes excess material, thereby determining the add-on level. High yarn tension during sizing elongates the warp sheet; excessive elongation reduces yarn extensibility and increases the likelihood of warp breakage in subsequent processes [9,10,11].

Ideally, sized yarn should exhibit increased tensile strength with minimal loss of elasticity to ensure good weaving performance [12,13]. Additionally, the weaver’s beam should be free from defects such as missing ends, crossed ends, lappers, and taped ends to guarantee smooth unwinding during weaving [14]. Warp stretch is another important consideration: although stretching can increase strength by about 15–20%, excessive size application reduces yarn extensibility, leading to higher warp breakage and reduced loom efficiency [15]. Hence, exceeding the optimal size add-on level adversely affects performance and increases yarn breakage.

One of the principal objectives of sizing is to enable the yarn to withstand abrasion and friction during shedding and reed beating. Abrasion resistance reflects the yarn’s ability to resist surface wear caused by repeated contact with loom components, and its evaluation provides a reliable indicator of weaving performance [16,17,18].

Yarn hairiness is another critical factor influencing weaving performance. Hairiness refers to the number and length of short fibers protruding from the yarn surface, which can lead to entanglement, friction, and pilling during weaving [19]. High hairiness increases the risk of yarn-to-yarn and yarn-to-loom contact, causing higher friction, increased abrasion, and a greater incidence of broken ends. Conversely, well-sized yarn with reduced hairiness provides smoother passage through the loom, decreases warp breakage, improves shed formation, and enhances overall weaving efficiency [20,21]. Therefore, controlling hairiness in combination with optimal size add-on is essential for achieving high-quality, high-performance woven fabrics.

Numerous studies have investigated the influence of sizing machine settings on the mechanical properties of sized warp yarns. However, previous research has generally evaluated individual responses separately and has not simultaneously considered the combined effects of multiple process parameters on size add-on, abrasion resistance, hairiness index, adhesion power, and elongation at break. Moreover, empirical relationships between the sizing variables and these responses have rarely been established using multivariate statistical approaches. To the best of our knowledge, the effect of sizing machine parameters on the hairiness index of sized warp yarn has not been systematically analyzed at this level of detail.

This study evaluates the weavability performance of cotton warp yarns sized with carboxymethyl cellulose derived from Saudi wheat straw (CMC_ws_), a low-cost and biodegradable sizing agent, under varying sizing process conditions. The research focuses on the influence of wet zone yarn tension, squeezing pressure, and sizing machine speed on key yarn properties, including size add-on, abrasion resistance, hairiness index, adhesion power, and elongation at break. Response surface methodology (Box–Behnken design) was employed to systematically investigate these effects. Detailed analyses, including ANOVA, regression modeling, and interaction effects visualized through 2D plots, were conducted to elucidate the impact of each process factor on the performance of CMC_ws_-sized yarns.

The originality of this research lies in the optimization of the sizing conditions of a newly developed biodegradable sizing agent, carboxymethyl cellulose derived from wheat straw (CMC_ws_), for application on cotton warp yarns. Derived from an abundant agricultural by-product, CMC_ws_ represents a sustainable and low-cost alternative to conventional petroleum-based sizing agents. This study systematically evaluates the effect of key sizing parameters on yarn mechanical performance and weavability efficiency, demonstrating the technical feasibility of CMC_ws_ and supporting its potential contribution to environmentally friendly and circular textile manufacturing.

## 2. Materials and Methods

### 2.1. Materials

Wheat straw, an agricultural residue, was obtained from a local farm in Tabuk, Saudi Arabia. The straw was first cut into small fragments and then dried in a convection oven at 50 °C for 24 h to minimize moisture content. Once dried, the material was ground using a kitchen blender to yield a fine powder, which was subsequently sieved, retaining only particles that passed through a 60-mesh screen for further processing.

All chemicals and reagents used in this study, including those for the synthesis and characterization of carboxymethyl cellulose derived from wheat straw (CMC_ws_), were sourced from Sigma-Aldrich (St. Louis, MO, USA) through a local supplier, SITEX (Ksar Hellal, Tunisia), and employed without additional purification. These included sodium hydroxide (≥98%), monochloroacetic acid (99%), 1-butanol (≥99.5%), and glycerol (≥98%). Distilled water was used in all preparation steps.

The degree of substitution (DS) of the synthesized carboxymethyl cellulose (CMC_ws_) attained a value of 1.23 at a monochloroacetic acid (MCA) concentration of 25 g per 10 g of dry cellulose, indicating a high level of carboxymethylation and hydrophilic character.

Correspondingly, the apparent viscosity (η) measured at room temperature and a shear rate of 300 s^−1^ increased to 903.03 cP, demonstrating the enhanced chain substitution and hydrophilicity of the resulting polymer.

The extracted cellulose exhibited a DP_v_ of approximately 933, corresponding to a viscosimetric molar mass of 151,200 g.mol^−1^.

The yarns investigated were cotton open-end warp yarns with a linear density of Nm 12.2 and a twist of 470 rpm, supplied by SITEX (Ksar Helal, Tunisia).

Each CMC_ws_ sizing formulation contained 6% of the primary sizing agent, supplemented with a plasticizer (glycerol, 8%) and a lubricant (Avirol, 7%). Glycerol was incorporated to improve the flexibility and workability of the size, while Avirol—a commercial lubricant composed of fatty acids, fatty alcohols, and emulsifiers—served to reduce friction between the yarns and weaving machinery components. These additives were included to enhance the functional performance of the sized cotton yarns during weaving [7,22].

### 2.2. Methods

The isolation and carboxymethylation of cellulosic fractions from Saudi wheat straw have been described in our previous work [7]. Cotton yarns were sized using a laboratory sizing machine (Karl Mayer-Rotal, Model SRL-SMR-SP) located at SITEX Tunisia Company to produce sized yarn samples. The machine was equipped with polyurethane squeezing rollers with a diameter of 120 mm and a hardness of 75 Shore A to ensure uniform pressure distribution and controlled size pick-up. In addition, the immersion roller had a diameter of 100 mm with an immersion depth of approximately 45 mm in the sizing bath to maintain stable yarn impregnation during the sizing process.

This study focused on evaluating the effects of wet zone yarn tension, sizing machine speed, and squeeze roller pressure on the performance of CMC_ws_-sized cotton yarns, while other factors—such as sizing agent concentration, viscosity, temperature, and pH—were kept constant (Table 1).

#### 2.2.1. Experimental Design and Statistical Analysis

Minitab 22 software, in combination with response surface methodology (RSM) and a Box–Behnken design, was used to plan the experiments and analyze the results. Three independent factors—wet zone yarn tension, machine speed, and squeezing pressure—were evaluated at three levels, while five response variables—adhesion power, elongation at break, size add-on, hairiness, and abrasion resistance—were measured. Factor levels are listed in Table 2. The software generated fifteen randomized experimental runs (Table 3), and fifteen corresponding yarn samples were produced.

Experimental results were analyzed using analysis of variance (ANOVA) at a 95% confidence level [23,24]. Model adequacy was assessed using Fisher’s F-test, as well as the coefficient of determination (R^2^), adjusted R^2^, predicted R^2^, and probability values (*p*-values) to evaluate the quality of the polynomial model fit.

#### 2.2.2. Characterization of Dependent Variables/Responses

- Yarn size add-on: Size add-on percentage is the amount of size materials added on the warp yarn surface [5,25]. In the current study, the desizing test method was used to determine the size add-on%. Using the following formula, the weights of the oven-dried sized and unsized yarn samples that were about 10 m long (three readings per sample) were used to determine the size add-on (%).
  (1) $$Size\;add\text{-}on\;\left(\%\right)=\frac{weight\;of\;sized\;yarn-weight\;of\;unsized\;yarn}{weight\;of\;unsized\;yarn}*100$$
- Adhesion power: The adhesive performance of the sizing formulation was assessed based on its ability to bind the individual fibers within the yarn structure. Unsized yarns, composed of loosely held fibers with minimal cohesion, exhibit lower tensile strength; thus, the increase in yarn strength following sizing provides a measure of the size’s bonding efficiency on the fiber surface. The breaking force and elongation at break of the sized yarns were measured using a Textechno Lloyd LR5K dynamometer (Textechno H. Stein GmbH & Co. KG, Monchengladbach, Germany) in accordance with ISO 2062 (AFNOR. NF G 06-037. 1990) [26]. A preliminary tension of 0.5 cN/tex was applied, and each test was carried out so that rupture occurred within 20 ± 3 s. To ensure statistical reliability, fifty replicates were tested for each yarn sample.
- Yarn abrasion resistance: Abrasion resistance reflects the ability of yarn to withstand surface wear caused by friction with loom components during weaving. No universally accepted standard exists for measuring yarn abrasion resistance; thus, testing is typically conducted following the manufacturer’s guidelines [27]. The propensity of warp yarns to break is closely linked to their abrasion resistance [28]. In this study, abrasion resistance was assessed using a Shirley yarn abrasion tester (SDL Atlas, Rock Hill, SC, USA). The instrument comprises two reciprocating bars: one made of hardened steel and the other covered with a standard abradant. Yarn specimens were secured between fixed and flexible holders, with an initial tension of 0.5 N applied to each yarn. Upon breakage, the flexible holder dropped, and the number of rubs (cycles) until failure was recorded. Ten replicates were tested per sample, and the mean value was used for analysis.
- Hairiness Index: Cotton warp yarns inherently contain irregularities, including protruding fiber ends, hooks, loops, gimlets, and other defects. Application of sizing reduces yarn hairiness, thereby lowering the risk of inter-yarn entanglement during weaving [14]. The hairiness of yarns, before and after sizing, was measured using a Shirley Electronic Yarn Hairiness Tester (SDL Atlas, Rock Hill, SC, USA) according to ASTM D5647 [7]. For each test, 50 m of yarn was evaluated at a travel speed of 30 m/min.

## 3. Results and Discussion

To evaluate the variation in yarn properties resulting from the influence of the three independent factors, wet zone yarn tension (*A*), squeezing pressure (*B*), and sizing machine speed (*C*), a 95% confidence level was employed. Factors with *p*-values below 0.05 were considered to have a statistically significant effect on the response variables, whereas those with *p*-values above 0.05 were deemed insignificant [29]. The adequacy of the regression models was further assessed using the coefficient of determination (*R*^2^), adjusted *R*^2^, and *F*-values. ANOVA results are summarized in Table 3, Table 4, Table 5, Table 6, Table 7 and Table 8.

**Figure 1:**
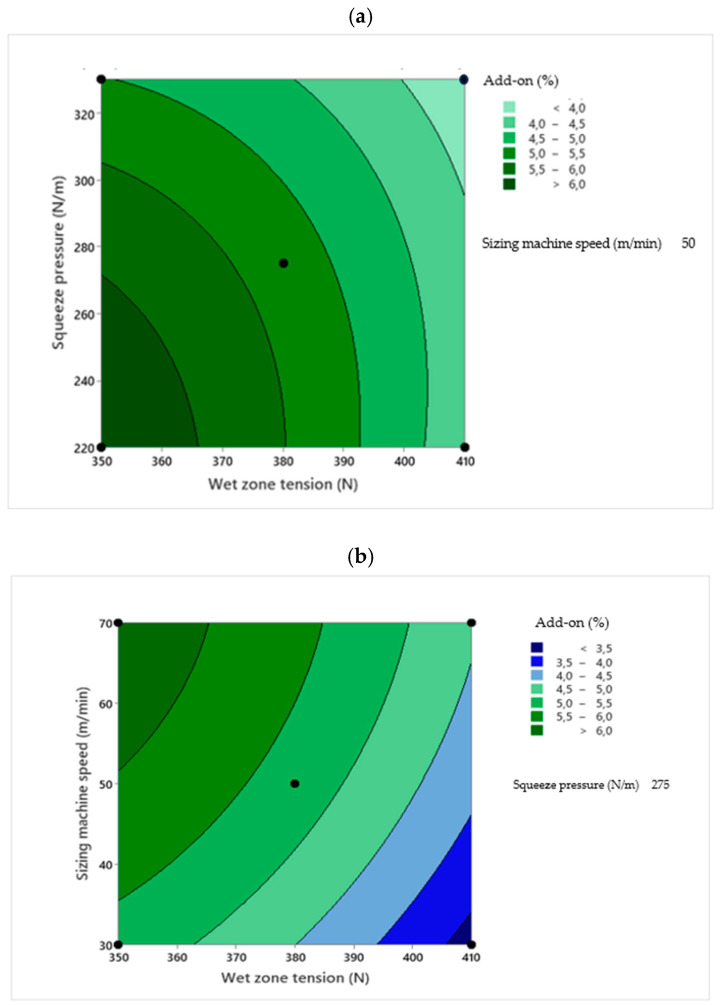

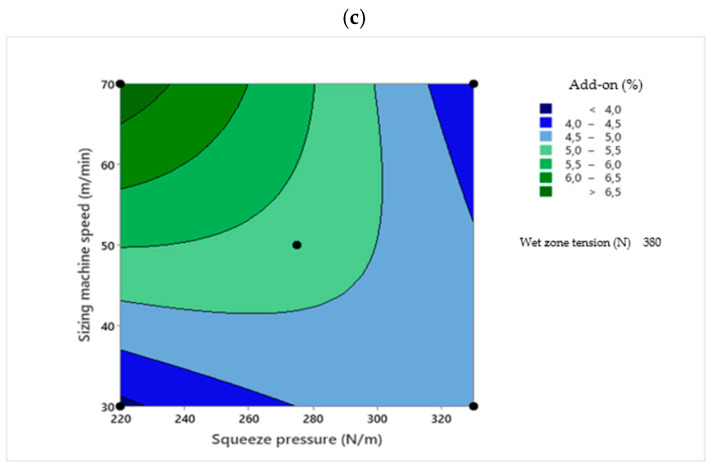
Contour plots showing the effect of wet zone yarn tension (**a**), squeezing pressure (**b**), and sizing machine speed (**c**) on CMC_ws_ size add-on%.

**Figure 2:**
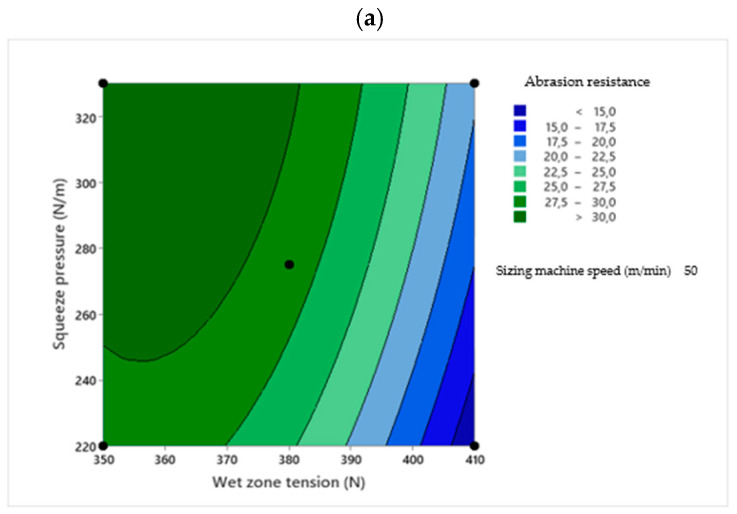

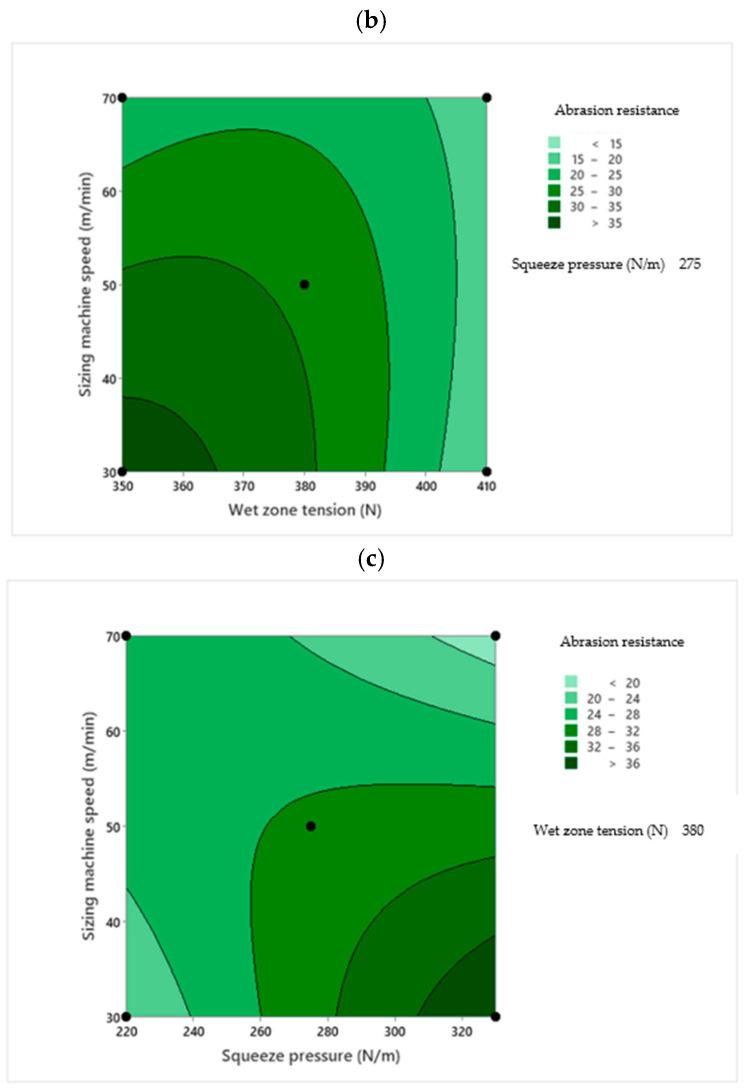
Contour plots showing the effect of wet zone yarn tension (**a**), squeezing pressure (**b**), and sizing machine speed (**c**) on CMC_ws_-sized yarn’s abrasion resistance.

**Figure 3:**
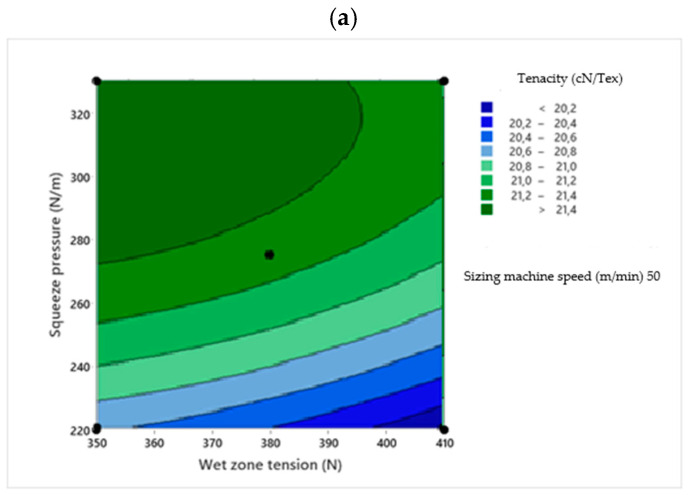

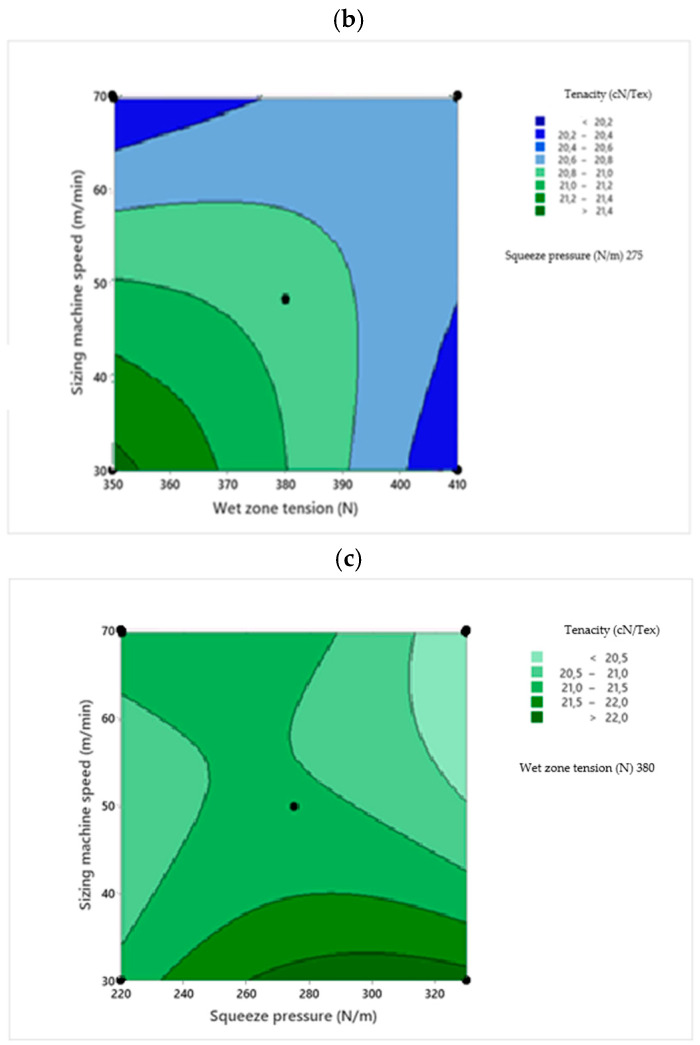
Contour plots showing the effect of wet zone yarn tension (**a**), squeezing pressure (**b**), and sizing machine speed (**c**) on CMC_ws_-sized yarn tenacity.

**Figure 4:**
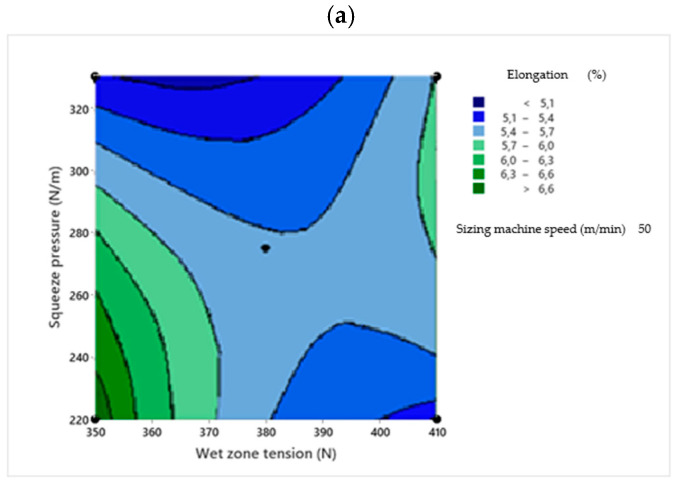

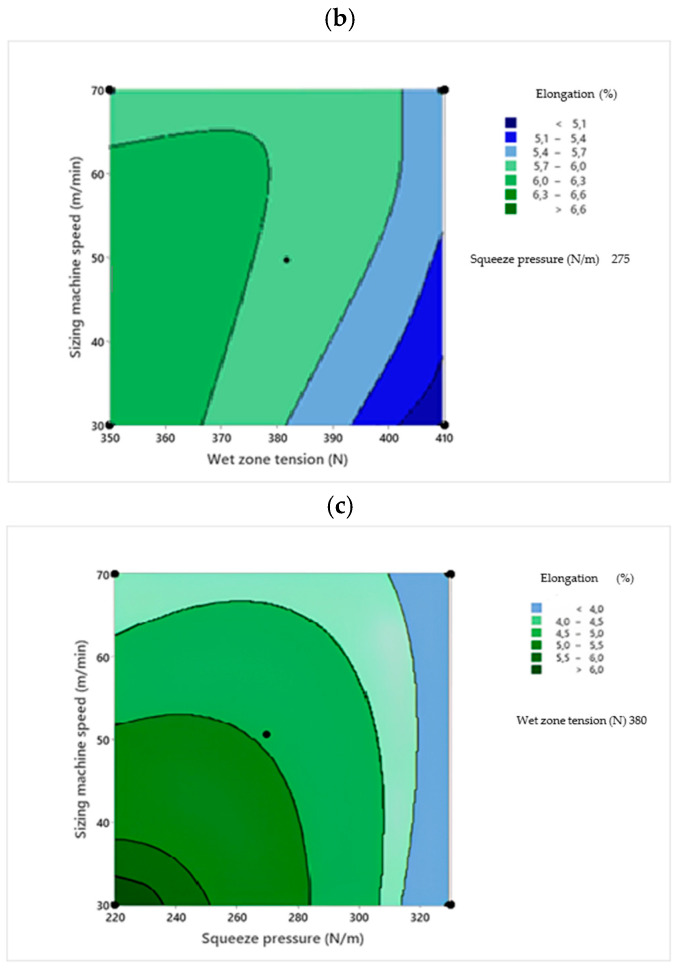
Contour plots showing the effect of wet zone yarn tension (**a**), squeezing pressure (**b**), and sizing machine speed (**c**) on CMC_ws_-sized yarn elongation at break.

**Figure 5:**
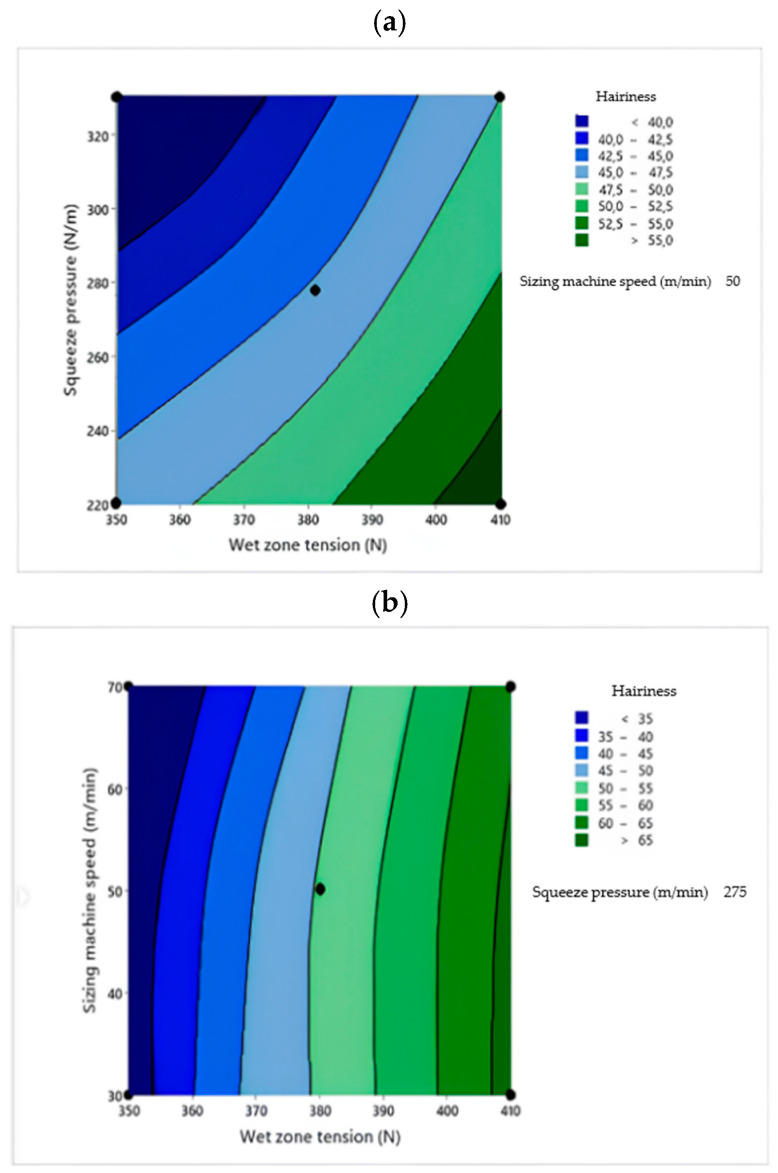

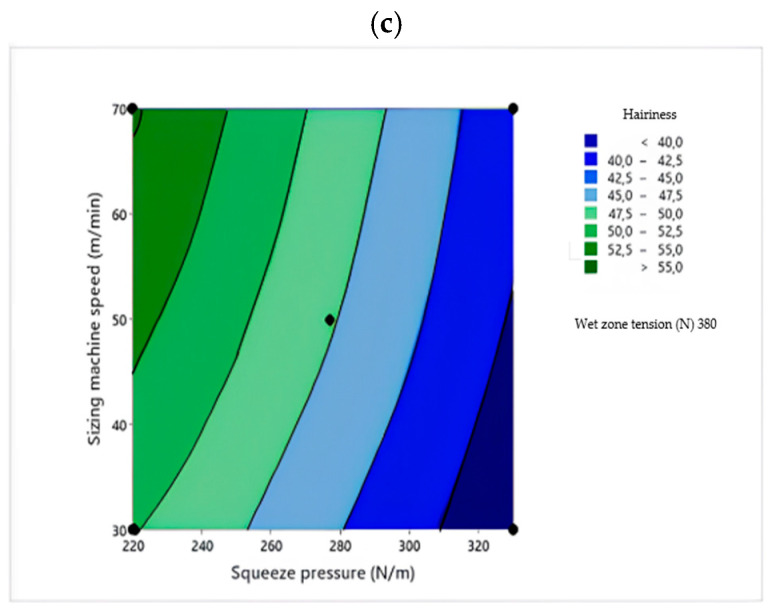
Contour plots showing the effect of wet zone yarn tension (**a**), squeezing pressure (**b**), and sizing machine speed (**c**) on CMC_ws_-sized yarn hairiness index.

Two-dimensional contour plots were used to visualize the relationship between the sizing process parameters and the response variables. Each plot illustrates the effect of two independent variables within their experimental ranges, while the third variable is held constant at its central value. The contour plots were generated based on the RSM regression models to identify the optimal process conditions [30]. Figure 1, Figure 2, Figure 3, Figure 4 and Figure 5 present the contour plots for different sizing performance parameters, including size add-on (%), adhesion power (cN/Tex), elongation at break (%), abrasion resistance (number of cycles), and hairiness index of warp yarns sized with Saudi wheat-straw-derived CMC_ws_.

### 3.1. Size Add-On Percentage

As presented in Table 4, the coefficient of determination (*R*^2^) for yarn size add-on is 99.36%, indicating an excellent agreement between the predicted and observed values. The corresponding adjusted *R*^2^ and predicted *R*^2^ values are 98.22% and 93.40%, respectively. The difference between the adjusted and predicted *R*^2^ values is less than 20%, demonstrating a strong correlation and confirming the reliability of the regression model in representing the actual behavior of yarn size add-on.

**Table 4:** Fit statistics for the characteristics of sized yarns.

Parameter	*R* ^2^	Adjusted *R*^2^	Predicted *R*^2^
Size Add-on	99.36%	98.22%	93.40%
Abrasion Resistance	97.86%	94.01%	94.05%
Breaking Force	97.24%	92.28%	84.37%
Elongation at Break	97.35%	94.80%	91.00%
Hairiness Index	91.73%	87.15%	86.86%

Table 5 shows the model is significant and leaner.

**Table 5:** Analysis of variance (ANOVA) for size add-on (%).

Source	df	Sum of Squares	Mean Square	*F*-Value	*p*-Value	
Model	9	14.9742	1.66380	86.77	<0.0001	Significant
*A*	1	6.7528	6.75281	195.06	<0.0001	
*B*	1	1.8915	1.89151	352.17	<0.0001	
*C*	1	2.5765	2.57645	98.64	<0.0001	
*A* ^2^	1	0.1767	0.17668	9.21	0.029	
*B* ^2^	1	0.1932	0.19321	10.08	0.025	
*C* ^2^	1	0.1351	0.13505	7.04	0.045	
*AB*	1	0.1980	0.19803	10.33	0.024	
*AC*	1	0.0196	0.01960	1.02	0.358	
*BC*	1	3.0976	3.09760	161.54	<0.0001	

The analysis of variance (ANOVA) shown in Table 5 confirms that the developed quadratic model is highly significant for predicting size add-on (%), with a model *F*-value of 86.77 and a *p*-value < 0.0001, indicating an excellent fit of the response surface model to the experimental data. All three main process parameters—wet zone yarn tension (*A*), squeeze pressure (*B*), and sizing machine speed (*C*)—exert statistically significant effects on size add-on (*p* < 0.0001). Among them, squeeze pressure exhibited the strongest influence, with the highest *F*-value (*F* = 352.17), followed by wet zone yarn tension (*F* = 195.06) and sizing machine speed (*F* = 98.64). This indicates that size add-on removal at the squeeze rollers is the dominant factor controlling size deposition, while yarn tension and machine speed also play critical but secondary roles. The quadratic terms *A*^2^, *B*^2^, and *C*^2^ were statistically significant (*p* < 0.05), confirming the presence of nonlinear effects and saturation behavior at extreme operating conditions. Interaction effects further highlight the coupled nature of the sizing process. In particular, the interaction between squeeze pressure and sizing machine speed (*BC*) was highly significant (*F* = 161.54, *p* < 0.0001), demonstrating that the positive effect of increasing machine speed on size add-on is strongly suppressed at higher squeeze pressures. The interaction between wet zone yarn tension and squeeze pressure (*AB*) was also significant (*p* = 0.024), indicating a combined influence of yarn tension and mechanical expression on size retention.

The regression model Equation (2) shows that the wet zone yarn tension, squeezing pressure, and the interaction of squeezing pressure with sizing machine speed have a negative correlation with size add-on%. But the linear term of sizing machine speed and the interactions between wet zone yarn tension and sizing machine speed have a positive correlation with the size add-on of the sized yarn.

Size Add-on (%) = 5.8 − 0.92 A − 0.74 B + 0.81 C − 0.18 A^2^ − 0.20 B^2^ − 0.16 C^2^ − 0.22 AB + 0.02 AC − 0.89 BC (2)

The 2D plots shown in Figure 1 illustrate the interaction effects of the independent variables on the size add-on (%). Response surface analysis revealed that sizing machine speed exerts the strongest positive influence on size add-on, whereas squeeze pressure has the most pronounced negative effect. A significant interaction between speed and pressure was observed, indicating that the positive impact of increased machine speed on size deposition diminishes at higher squeeze pressures. Wet zone tension was found to play a secondary but consistent role, with higher tension reducing size retention.

Figure 1a shows the contour plot of wet zone tension versus squeeze pressure, revealing a steady decline in size add-on with increasing levels of both variables. This trend confirms that the combined action of yarn tension and mechanical squeezing promotes the removal of size from the yarn surface. Figure 1b presents the interaction between wet zone tension and sizing machine speed, where the nearly parallel contour lines indicate a weak interaction between these parameters. This suggests that the enhancing effect of higher machine speed on size pick-up is largely independent of yarn tension within the studied range. In contrast, Figure 1c illustrates the squeeze pressure–sizing machine speed interaction, characterized by pronounced curvature and closely spaced contours, indicating a strong coupling between squeezing intensity and residence time. At low squeeze pressure, increasing machine speed significantly improves size add-on due to shorter squeeze-out time and greater size retention. However, at higher squeeze pressures, the dominant squeezing action suppresses the beneficial influence of speed, leading to reduced add-on even at elevated machine speeds.

The significance of the quadratic terms in the ANOVA confirms curvature in the response surface, indicating that the size add-on varies non-linearly with the processing variables and exhibits a distinct optimum region. Combined contour analysis identifies this optimum at relatively low squeeze pressure, moderate wet zone yarn tension, and high sizing machine speed, conditions that maximize size deposition while minimizing squeeze-out and tension-induced depletion. Specifically, the maximum size add-on was achieved at a sizing machine speed of 70 m/min, and squeeze pressure of 220 N/m with wet zone tension maintained at 380 N. Conversely, the lowest add-on occurs at high squeeze pressure, high tension, and low machine speed, where prolonged squeezing and yarn stretching jointly remove a substantial fraction of the applied size. Under these conditions, penetration into the yarn structure is limited while surface coating efficiency remains high, consistent with previous reports [31,32]. ANOVA and contour analyses consistently demonstrate that size add-on is governed primarily by the coupled squeeze–time mechanism defined by squeeze pressure and sizing machine speed, while wet zone yarn tension modulates retention through its influence on yarn compaction and liquid accessibility. These interactions explain the observed response surface behavior and provide a quantitative basis for optimizing sizing parameters to achieve maximum size add-on and improved warp protection.

### 3.2. Yarn Abrasion Resistance

In the current research, the effect of wet zone yarn tension, squeezing pressure, and speed of sizing machine on the abrasion resistance of sized yarn is analyzed.

Fit statistics in Table 4 indicate that the values of adjusted *R*^2^ and predicted *R*^2^ for yarn abrasion resistance are found to be 94.01% and 94.05% respectively. This indicates a high degree of correlation between the actual and predicted values.

As observed from Table 6, the model is significant with a *p*-value of 0.001. The linear term of wet zone yarn tension with (*p*-value < 0.0001) has the largest effect on the yarn abrasion resistance, followed by linear term of sizing machine speed with *p*-values of 0.003 and the interaction effect of wet zone yarn tension and sizing machine speed (*p*-value = 0.006), quadratic term of wet zone yarn tension (*p*-value = 0.0007), the interaction effect of squeeze pressure and sizing machine speed (*p*-value = 0.001), and linear term of squeezing pressure with *p*-values of 0.016.

**Table 6:** Analysis of variance (ANOVA) for sized yarn abrasion resistance.

Source	df	Sum of Squares	Mean Square	*F*-Value	*p*-Value	
Model	9	887.517	98.613	25.39	0.001	Significant
*A*	1	351.125	351.125	90.42	<0.0001	
*B*	1	50.000	50.000	12.88	0.016	
*C*	1	105.125	105.125	27.07	0.003	
*A* ^2^	1	77.564	77.564	19.97	0.007	
*B* ^2^	1	2.564	2.564	0.66	0.453	
*C* ^2^	1	9.256	9.256	2.38	0.183	
*AB*	1	6.250	6.250	1.61	0.260	
*AC*	1	81.000	81.000	20.86	0.006	
*BC*	1	210.250	210.250	54.14	0.001	

The quadratic regression model illustrated in Equation (3) shows that the wet zone yarn tension, sizing machine speed, the quadratic term of wet zone yarn tension, and the interaction of squeezing pressure and sizing machine speed have a negative correlation with the yarn abrasion resistance. But the squeezing pressure has a positive correlation with the sized yarn abrasion resistance.

Based on the coefficient of the independent variables of the regression equation, the following can be claimed:

- For each unit increase in the number of wet zone yarn tension, with other independent variables being constant, the yarn abrasion resistance is reduced by 3.45%.
- For each unit force increase in the squeezing pressure, with other independent variables being constant, the yarn abrasion resistance is increased by 1.15%.
- For each unit of sizing machine speed increases in the sizing process, with other independent variables being constant, the yarn abrasion resistance is reduced by 1.95%, and the other relations can be explained in a similar manner from Equation (3).

The effects of wet zone yarn tension (*A*), squeezing pressure (*B*), and sizing machine speed (*C*) on yarn abrasion resistance were evaluated using response surface methodology, yielding the following regression model:

Abrasion resistance (No of cycles) = 28.6 − 3.45 A + 1.15 B − 1.95 C − 1.60 A^2^ + 025 B^2^ − 0.75 C^2^ + 0.40 AB + 1.25 AC − 2.85 BC (3)

The interaction effects of wet zone yarn tension, squeezing pressure, and sizing machine speed on yarn abrasion resistance are presented in Figure 2.

Figure 2a shows the interaction between wet zone tension and squeeze pressure at a fixed sizing machine speed. Abrasion resistance declines with increasing wet zone tension because higher yarn tension stretches the yarn and causes the sizing material to migrate toward the surface, making it easier to remove during abrasion. At moderate wet zone tension, increasing squeeze pressure improves the penetration of the sizing into the yarn, strengthening inter-fiber bonding and enhancing abrasion performance.

Figure 2b illustrates the interaction between wet zone tension and sizing machine speed at constant squeeze pressure. Higher wet zone tension reduces abrasion resistance, while increasing sizing machine speed limits the time available for sizing penetration, leaving more material on the surface and decreasing abrasion resistance. The higher tension stretches the warp yarn, moving size from the core to the surface [17], so the outer layer is more easily worn away during abrasion, reducing the number of cycles the yarn can endure and increasing the likelihood of breaking during weaving. Figure 2c presents the combined effect of squeeze pressure and sizing machine speed at a fixed wet zone tension. Abrasion resistance improves with higher squeeze pressure and moderate sizing machine speed. At lower machine speeds and higher squeezing pressures, the increased residence time and compression facilitate deeper penetration of CMC_ws_ molecules into the yarn structure. The flexible polymer chains of CMC_ws_ allow the sizing agent to uniformly coat the fibers and fill inter-fiber spaces, resulting in improved fiber cohesion and reduced yarn hairiness. In addition, the cohesive nature of the formed CMC_ws_ film contributes to enhanced abrasion resistance and tensile performance by forming a continuous and protective layer around the yarn. Conversely, higher sizing machine speed reduces abrasion resistance because, although more size is deposited at faster speeds, the reduced squeezing time limits penetration into the yarn core, leaving the size mainly on the surface, in line with findings by Fernando et al. 2015 [31].

The optimal conditions for maximum abrasion resistance were identified at a wet zone yarn tension of about 340 N, squeezing pressure near 330 N/m, and sizing machine speed around 45 m/min. This combination aligns with the peak abrasion-resistance region in the response surfaces. Enhanced performance is associated with deeper size penetration and stronger inter-fiber anchorage under high squeezing pressure and moderate tension.

### 3.3. Yarn Adhesion Power and Elongation at Break

In Table 7, model adequacy was evaluated through analysis of variance (ANOVA) of sized yarn tenacity, which confirmed the statistical significance of the fitted model (*p* = 0.002), indicating that the model adequately represents the experimental data.

**Table 7:** Analysis of variance (ANOVA) for CMC_ws_-sized yarn tenacity and elongation.

Source	df	Sum of Squares	Mean Square	*F*-Value	*p*-Value	
ANOVA for Sized yarn Tenacity (cN/Tex)						
Model	9	6.58935	0.73215	19.59	0.002	Significant
*A*	1	0.58861	0.58861	50.36	0.011	
*B*	1	0.06301	0.06301	15. 75	0.001	
*C*	1	1.88180	1.88180	1.69	0.051	
*A* ^2^	1	0.03245	0.03245	38.87	0.034	
*B* ^2^	1	0.85514	0.85514	22.89	0.005	
*C* ^2^	1	1.29074	1.29074	34.54	0.002	
*AB*	1	0.25503	0.25503	6.83	0.048	
*AC*	1	0.12960	0.12960	3.47	0.122	
*BC*	1	1.32250	1.32250	35.39	0.002	
ANOVA for Sized yarn Elongation at break (%)						
Model	9	4.62	0.513	38.9	<0.0001	Significant
*A*	1	1.48	1.48	112.4	<0.0001	
*B*	1	0.81	0.81	61.5	0.0002	
*C*	1	0.36	0.36	27.4	0.0021	
*A* ^2^	1	0.89	0.89	67.6	0.0002	
*B* ^2^	1	0.74	0.74	56.1	0.0003	
*C* ^2^	1	0.21	0.21	15.9	0.0092	
*AB*	1	0.69	0.69	52.3	0.0004	
*AC*	1	0.42	0.42	31.9	0.0016	
*BC*	1	0.26	0.26	19.7	0.0061	

The quantitative relationships between variables, wet zone yarn tension, squeeze pressure, and sizing machine speed and their interactions with the response values are described by the regression model Equation (4):

Tenacity (cN/Tex) = 21.05 − 0.22 A + 0. 12 B − 0.045 C − 0.011 A^2^ + 0.042 B^2^ − 0.0045 C^2^ + 0.019 AB − 0.016 BC (AC omitted due to insignificance, p = 0.122)(4)

A regression equation was produced by the Minitab 22 program software in terms of coded parameters for significant factors. It demonstrates a negative relation between the strength of the sized yarn and the wet zone yarn tension, interactions between squeeze pressure and sizing machine speed, and the quadratic terms of wet zone yarn tension and sizing machine speed. Nonetheless, there is a positive association between the sized yarn strength and the squeezing roller pressure, as well as the interactions between wet zone yarn tension and squeeze pressure and the quadratic term of squeeze pressure.

The interaction effects of wet zone yarn tension, squeezing pressure, and sizing machine speed on average-sized yarn strength are illustrated in Figure 3 using 2D contour plots. The statistically significant quadratic model (*F* = 19.59, *p* = 0.002) is visually supported by these plots, which highlight the dominant influence of wet zone yarn tension and squeezing pressure over sizing machine speed in controlling yarn tenacity.

As shown in Figure 3a, the wet zone yarn tension–squeezing pressure contour plot displays steep gradients along the wet zone yarn tension axis, consistent with the high ANOVA F-value for wet zone yarn tension (*F* = 50.36). Tenacity initially shows a slight increase but then declines sharply with rising wet zone yarn tension, reflecting the negative linear and quadratic coefficients in the model. This reduction is primarily due to fiber slippage within the yarn structure under high tension. In polyester–cotton blend yarns with low fiber uniformity, increasing tension decreases fiber-to-fiber friction, reducing the yarn’s ability to resist applied loads, in agreement with previous studies [8]. In contrast, moderate increases in squeezing pressure enhance tenacity, as evidenced by the broad high-tenacity plateau at intermediate pressures. The elliptical contour pattern confirms the statistically significant wet zone yarn tension–squeezing pressure interaction (*F* = 6.83, *p* = 0.048), indicating that the positive effect of squeezing pressure is most pronounced at lower tension levels.

Figure 3c highlights the squeezing pressure–sizing machine speed interaction, which has the strongest impact on yarn strength. The contour plot shows pronounced distortion and tightening at high squeezing pressure and SMS values, corresponding to the significant squeezing pressure × sizing machine speed interaction (*F* = 35.39, *p* = 0.002). Higher squeezing pressure promotes deeper penetration of sizing into the yarn core, increasing inter-fiber binding and yarn compactness, which enhances fiber cohesion and reduces slippage, consistent with [7,18,33]. While sizing machine speed alone has a relatively minor effect, increasing machine speed under high squeezing pressure markedly reduces tenacity due to insufficient residence time for size fixation combined with mechanical compression.

At a squeezing roller pressure of 330 N/m, a sizing machine speed of 30 m/min, and a constant wet zone yarn tension of 380 N, the tensile strength reached a maximum level. That means when the sizing machine speed is at its lowest level, the squeezing pressure should be at its highest level to obtain the maximum tensile strength of the yarn. This is because at low speed, the yarn waiting time in the sizing box is greater, and there is enough time for the size material to penetrate the core of the yarn, and higher pressure also assists the penetration of size material into the core of the yarn. The cohesive characteristics of sizing agents are essential for protecting the yarn surface during weaving. This behavior is generally evaluated through film strength, which represents the internal cohesion of the sizing material and its ability to create a strong and durable protective film around the yarn. The CMC_ws_-sizing agent exhibited high film strength, demonstrating its capability to form an effective and cohesive coating on the yarn surface. Such performance indicates that CMC_ws_ can improve weavability by increasing fiber cohesion while reducing yarn hairiness and breakage during weaving. Therefore, a CMC_ws_-based sizing agent possessing strong cohesive properties and high adhesion to the fiber surface is expected to enhance weaving performance and overall weaving efficiency [7].

Table 4 shows that the values of adjusted *R*^2^ and predicted *R*^2^ for elongation at break are found to be 94.80% and 91.00%, respectively. This indicated a high degree of correlation between the actual and predicted values. Similarly, the difference between the adjusted *R*^2^ and predicted *R*^2^ is less than 20%, which means there is no over-fitting and a regression model can properly predict the responses.

In Table 7, model adequacy was evaluated through analysis of variance (ANOVA) of elongation at break, which confirmed the statistical significance of the fitted model (*p* < 0.0001), indicating that the model adequately represents the experimental data.

The analysis of variance confirms that the developed quadratic model for sized yarn elongation at break is highly significant (*F* = 38.9, *p* < 0.0001), indicating that the selected processing variables adequately explain the observed response. Among the linear terms, wet zone yarn tension exhibits the strongest effect on elongation (*F* = 112.4, *p* < 0.0001), identifying it as the dominant controlling parameter. Squeeze pressure also shows a strong and statistically significant influence (*F* = 61.5, *p* = 0.0002), while sizing machine speed has a moderate but significant effect (*F* = 27.4, *p* = 0.0021).

The significance of the quadratic terms *A*^2^ (*F* = 67.6, *p* = 0.0002) and *B*^2^ (*F* = 56.1, *p* = 0.0003) reveals pronounced nonlinearity in the elongation response, which is consistent with the curvature observed in the contour plots. Although smaller in magnitude, the quadratic effect of *C*^2^ remains statistically significant (*F* = 15.9, *p* = 0.0092), indicating diminishing returns in elongation at higher machine speeds.

Interaction effects are also significant, confirming the coupled influence of processing variables on elongation behavior. The *AB* interaction (*F* = 52.3, *p* = 0.0004) is particularly strong, explaining the saddle-shaped contours observed when these factors are varied simultaneously. Significant interactions between *AC* (*F* = 31.9, *p* = 0.0016) and *BC* (*F* = 19.7, *p* = 0.0061) further demonstrate that the effect of sizing speed depends on the applied tension and squeeze pressure.

Figure 4 and Equation (5) show that the elongation response is governed primarily by wet zone yarn tension, with squeeze pressure exerting a strong secondary influence. Significant quadratic and interaction effects confirm the nonlinear and coupled nature of mechanical stresses during sizing, validating the suitability of the second-order response surface model:

Elongation (%) = 5.52 − 0.42 A − 0.31 B + 0.24 C − 0.38 A^2^ − 0.27 B^2^ − 0.12 C^2^ − 0.29 AB + 0.21 AC − 0.18 BC (5)

The negative coefficients associated with wet zone yarn tension and squeeze pressure confirm their adverse effect on elongation, whereas the positive linear coefficient of sizing machine speed indicates a beneficial influence within the investigated range. The significant quadratic and interaction terms demonstrate the nonlinear response of elongation to the combined tensile and compressive stresses imposed during sizing. In particular, the significant *AB* and *AC* interactions emphasize the coupled influence of tension and compression on yarn extensibility.

Figure 4a demonstrates that yarn elongation declines substantially when high wet zone yarn tension is combined with high squeeze pressure, whereas lowering the squeeze pressure enhances elongation, particularly at lower tension levels. The saddle-shaped contour pattern indicates a pronounced interaction between these two parameters. Elevated squeeze pressure intensifies yarn compaction and size penetration, resulting in increased stiffness and reduced extensibility; this effect is amplified under high wet zone yarn tension. Therefore, simultaneous application of high tension and high pressure should be avoided when elongation retention is required. In agreement with earlier reports [8,34], increasing wet zone yarn tension significantly contributes to elongation loss in sized yarns.

Figure 4b depicts the interaction between squeeze pressure and sizing machine speed. The highest elongation values are observed at low squeeze pressure combined with low to moderate machine speeds. Increasing squeeze pressure causes a consistent reduction in elongation that is largely independent of machine speed, indicating that pressure exerts the dominant influence. Excessive squeezing leads to over-densification and diminished yarn elasticity, whereas moderate speeds favor more uniform size distribution without severe compaction, thereby preserving flexibility. Hence, at constant wet zone yarn tension, squeeze pressure emerges as the principal factor governing elongation.

Figure 4c shows that elongation decreases progressively with increasing wet zone yarn tension, confirming the detrimental effect of excessive wet-state tensile loading on yarn extensibility. Higher elongation is achieved at low to moderate tension, especially at moderate-to-high sizing machine speeds. Beyond approximately 390–400 N, elongation declines sharply and becomes relatively insensitive to machine speed, demonstrating the dominant role of tension in this region. This suggests that increasing machine speed can partially compensate for elongation loss at moderate tension by shortening wet residence time, although this benefit diminishes under high-tension conditions.

Model optimization predicts maximum elongation at approximately 360 N wet zone yarn tension, 250 N/m squeeze pressure, and 60 m/min machine speed, corresponding to a predicted elongation of about 6.0%, consistent with the peak region observed in the contour plots. The comparatively wide optimum region indicates good process robustness, with only minor variation in elongation within this operating window. These optimized conditions align with the underlying physical mechanisms: lower wet zone tension reduces excessive wet stretching and preserves extensibility after drying; moderate squeeze pressure avoids over-compaction and stiffness; and higher machine speed shortens wet exposure time, limiting mechanical damage while promoting more uniform size distribution.

### 3.4. Yarn Hairiness

Table 8 shows that the model is significant and leaner. The interaction effects of wet zone yarn tension (*A*), squeeze pressure (*B*), and sizing machine speed (*C*) on yarn hairiness were evaluated using response surface methodology.

**Table 8:** Analysis of variance (ANOVA) for CMC_ws_-sized yarn hairiness index.

Source	df	Sum of Squares	Mean Square	*F*-Value	*p*-Value	
Model	9	182.64	20.29	28.47	<0.0001	Significant
*A*	1	96.52	96.52	135.41	<0.0001	
*B*	1	38.14	38.14	53.52	0.0002	
*C*	1	21.87	21.87	30.67	0.0009	
*A* ^2^	1	11.36	11.36	15.93	0.0053	
*B* ^2^	1	8.94	8.94	12.54	0.0094	
*C* ^2^	1	4.62	4.62	6.48	0.038	
*AB*	1	0.86	0.86	1.21	0.31	
*AC*	1	0.51	0.51	0.71	0.43	
*BC*	1	0.82	0.82	1.15	0.32	

The quadratic model was highly significant (*F* = 28.47, *p* < 0.0001), confirming model adequacy. The coefficient of determination was high (*R*^2^ = 91.73%), the adjusted *R*^2^ (87.15%) and predicted *R*^2^ (86.86%) values were in close agreement, indicating strong predictive capability.

The quadratic regression model relating the hairiness index to the sizing process variables in actual units is expressed as follows (Equation (6)):

Hairiness Index = 45.2 + 0.82 A − 0.54 B + 0.61 C + 0.21 A^2^ + 0.12 B^2^ + 0.18 C^2^ − 0.24 AB + 0.19 AC − 0.16 BC (6)

The positive coefficients of wet zone yarn tension and sizing machine speed indicate that increasing yarn tension and machine speed increase fiber protrusion and surface irregularity, thereby increasing hairiness. In contrast, the negative coefficient of squeeze pressure confirms that higher squeezing pressure improves size penetration and inter-fiber binding, reducing protruding fibers. The significant interaction terms demonstrate that compressive forces can partly counteract tension-induced fiber emergence, whereas the combined action of high tension and high speed exacerbates hairiness.

Figure 5a shows the wet zone yarn tension–squeeze pressure interaction at constant sizing machine speed. Hairiness increases markedly with wet zone yarn tension, as evidenced by the steep contour gradients along the tension axis. Increasing squeeze pressure reduces hairiness, particularly at moderate tension levels, indicating that enhanced compression improves fiber binding. The tilted contours confirm a significant wet zone yarn tension–squeeze pressure interaction, demonstrating that the beneficial effect of squeeze pressure is more pronounced at lower tension.

Figure 5b illustrates the wet zone yarn tension–sizing machine speed interaction at constant squeeze pressure. Hairiness increases with both wet zone yarn tension and sizing machine speed, with maximum values occurring at high tension and high speed. Higher machine speeds reduce the residence time for size penetration, leaving more material on the yarn surface and allowing fiber ends to protrude. The interaction contours indicate that the detrimental effect of machine speed becomes stronger at elevated tension.

Figure 5c presents the squeeze pressure–sizing machine speed interaction at constant wet zone yarn tension. Hairiness decreases with increasing squeeze pressure but increases with sizing machine speed. At high squeezing pressure, hairiness remains relatively low even at moderate speeds, showing that compression partially compensates for reduced penetration time. Conversely, the combination of low squeeze pressure and high sizing machine speed produces the highest hairiness due to insufficient size fixation and weak fiber anchorage.

Optimization results indicated that minimum yarn hairiness is attained at a wet zone yarn tension of approximately 350 N, squeezing pressure around 320 N/m, and sizing machine speed near 50 m/min. Within this operating region, the predicted hairiness index was about 42, corresponding to the lowest zone identified in the contour plots. The relatively broad optimum suggests that hairiness is only weakly sensitive to small parameter variations. Lower wet zone tension reduces fiber protrusion, moderate-to-high squeezing pressure improves fiber binding through deeper size penetration, and moderate machine speed ensures adequate fixation without excessive surface accumulation.

## 4. Conclusions

The sizing process plays a decisive role in determining the quality and weavability of warp yarns using Saudi wheat-straw-derived CMC_ws_; therefore, achieving maximum weaving efficiency requires optimized sizing conditions. The present results demonstrate the technical feasibility of CMC_ws_ and support its potential contribution to environmentally friendly and circular textile manufacturing.

Regression analysis shows that increasing wet zone yarn tension leads to a decrease in both size add-on and abrasion resistance of the sized yarns. In contrast, increasing squeezing pressure significantly improves abrasion resistance while reducing size add-on percentage due to enhanced mechanical expression of the size from the yarn surface. Furthermore, higher sizing machine speed promotes greater size retention on the yarn, resulting in increased size add-on, whereas abrasion resistance decreases because reduced residence time limits penetration of the size into the yarn core.

In addition to these responses, the mechanical properties of the sized yarn were also affected by the process parameters. Yarn tenacity and breaking elongation decreased with increasing wet zone yarn tension and sizing machine speed, indicating that excessive stretching and insufficient size penetration weaken fiber cohesion within the yarn structure. Conversely, squeezing pressure exhibited a positive effect on both tenacity and elongation, as improved size compaction enhances inter-fiber bonding and load distribution. Yarn hairiness showed an opposite trend, increasing with higher wet zone yarn tension and sizing machine speed due to surface fiber protrusion caused by tension-induced fiber migration and reduced coating uniformity, while increasing squeezing pressure effectively reduced hairiness through better fiber binding and surface consolidation.

Beyond individual factor effects, significant interaction effects were also observed. In particular, the interaction between squeezing pressure and sizing machine speed exerted a pronounced influence on abrasion resistance, indicating that the beneficial effect of high squeezing pressure is maximized at moderate machine speeds. Similar interaction tendencies were observed for tenacity and hairiness, confirming that optimal mechanical performance requires a balanced combination of compaction and penetration conditions during sizing.

The regression models confirmed that wet zone yarn tension and squeezing pressure exhibit negative correlations with size add-on percentage, whereas sizing machine speed shows a positive correlation with size add-on. Conversely, wet zone yarn tension and sizing machine speed have negative correlations with yarn abrasion resistance, tenacity, and breaking elongation, while squeezing pressure has a positive correlation with these properties. For yarn hairiness, wet zone yarn tension and sizing machine speed showed positive correlations, whereas squeezing pressure exhibited a negative correlation.

Maximum size add-on occurred at a sizing machine speed of 70 m/min and squeezing pressure of 220 N/m with wet zone yarn tension fixed at 380 N. The optimum conditions for abrasion resistance were about 340 N wet zone tension, 330 N/m squeezing pressure, and 45 m/min speed, whereas maximum yarn tenacity was reached at 330 N/m, 30 m/min, and 380 N. Elevated elongation values were associated with a wet zone yarn tension of 360 N and sizing machine speed of 60 m/min. Numerical optimization predicted the lowest hairiness at approximately 350 N tension, 320 N/m pressure, and 50 m/min speed.

## Figures and Tables

**Table 1:** Composition and preparation parameters of size formulations.

Parameter	Value
Size concentration, %	6
Plasticizer concentration, %	8
Lubricant concentration, %	7
Viscosity, cP	60–200
Mixing temperature, °C	90
pH	7.0
Duration, h	2

**Table 2:** Selected factors and associated levels.

Factor	Lower Level	Average	Higher Level
Wet zone yarn tension (*A*)	350 N	380 N	410 N
Squeezing pressure (*B*)	220 N/m	275 N/m	330 N/m
Sizing machine speed (*C*)	30 m/min	50 m/min	70 m/min

**Table 3:** Average responses of CMC_ws_-sized yarn properties under different factor combinations.

Factors					Responses			
	1	2	3	1	2	3	4	5
Run	Wet Zone Yarn Tension (N)	Squeezing Pressure (N/m)	Sizing Machine Speed (m/min)	Size Add-On (%)	Abrasion Resistance (No of Cycle	Tenacity (cN/Tex)	Elongation (%)	Hairiness Index
3	350	330	50	5.01	31	20.13	5.28	48.3
13	380	275	50	5.26	32	21.07	6.58	49.0
10	380	330	30	4.57	40	19.85	5.78	38.7
15	380	275	50	5.12	28	21.13	6.46	47.5
8	410	275	70	4.46	17	22.19	5.84	30.5
5	350	275	30	5.38	37	21.00	5.75	42.5
6	410	275	30	3.32	15	21.80	5.84	64.7
9	380	220	30	3.84	20	21.36	4.11	56.3
14	380	275	50	5.40	26	21.07	5.31	39.5
4	410	330	50	3.70	20	21.28	5.17	65.0
7	350	275	70	6.24	21	22.11	4.92	60.4
2	410	220	50	4.17	13	20.77	6.96	54.7
11	380	220	70	6.87	27	21.40	4.99	44.0
12	380	330	70	4.08	18	22.19	4.04	47.1
1	350	220	50	6.37	29	20.63	5.28	56.3

## Data Availability

The original contributions presented in this study are included in the article. Further inquiries can be directed to the corresponding author.
